# Clinical, electrocardiographic, echocardiographic, and angiographic predictors for the final infarct size assessed by cardiac magnetic resonance in acute STEMI patients after primary percutaneous coronary intervention

**DOI:** 10.1186/s43044-024-00526-x

**Published:** 2024-08-24

**Authors:** Ahmad Samir, Sherif Nagy, Magdy Abdelhamid, Hossam Kandil

**Affiliations:** Faculty of Medicine, Cairo University, Cairo, Egypt

**Keywords:** Infarct size, STEMI, Cardiac magnetic resonance, Global longitudinal strain, Residual ST segment elevation

## Abstract

**Background:**

Final infarct size (IS) after ST segment elevation myocardial infarction (STEMI) is a major predictor of mortality. Seeking early predictors for final IS can guide individualized therapeutic strategies for those recognized to be at higher risk.

**Results:**

Eighty STEMI patients successfully treated with primary percutaneous coronary intervention (pPCI) underwent baseline (within 48 h) 2D, 3D echocardiography with speckle tracking and then underwent cardiac magnetic resonance (CMR) at 3 months to assess the final IS. After recruitment, 4 patients were excluded for uncontainable claustrophobia while 76 patients completed the final analysis. The mean ± standard deviation age was 54.1 ± 10.9 years, 84% were males, 25% had diabetes, 26% were hypertensives, 71% were current smokers, 82% had dyslipidemia, and 18% had a family history of premature coronary artery disease. By 3 months, CMR was performed to accurately evaluate the final IS. In univariate regression analysis, the admission heart rate, baseline and post-pPCI ST elevation, STEMI location (anterior vs. inferior), highest peri-procedural troponin, large thrombus burden, baseline thrombolysis in myocardial infarction flow grade, the final myocardial blush grade, the 2D and 3D left ventricular ejection fraction (LVEF), and the 2D and 3D global longitudinal strain (GLS) parameters were significant predictors for the final IS. In the multivariate regression analysis, four models were constructed and recognized the residual post-PCI ST segment elevation, the highest peri-procedural troponin, the 2D-LVEF, 3D-LVEF, and 2D-GLS as significant independent predictors for final IS.

**Conclusions:**

In STEMI patients who underwent successful pPCI, early predictors for the final IS are vital to guide therapeutic decisions. The residual post-pPCI ST elevation, the highest peri-procedural troponin, and the baseline 2D-LVEF, 3D-LVEF, and 2D-GLS can be excellent and timely tools to predict the final IS.

## Background

ST segment elevation myocardial infarction (STEMI) and its sequelae remain among the leading causes of death and disability worldwide [1]. The main goal in the management of acute myocardial infarction (AMI) is the timely restoration of flow in the infarct-related artery (IRA) to maximize myocardial salvage. Primary percutaneous coronary intervention (pPCI) has proved to be superior to thrombolytic reperfusion in terms of reducing mortality and preserving left ventricular (LV) function [2]. Mortality is considered the best parameter for evaluating the efficacy of reperfusion after AMI [3]. However, utilizing this hard endpoint with few event rates necessitates a large sample and extended years of follow-up to assess the impact of any novel advents added to the already effective contemporary reperfusion practices. Hence, other markers of reperfusion efficacy are conveniently needed. Among these, final infarct size (IS) has often been used as a surrogate for reperfusion efficacy and was found to be well correlated with mortality [4].

Notably, the final IS is influenced by several factors including the extent of myocardium supplied by the IRA (shaping the area at risk (AAR)), the residual flow to the ischemic territory (the collateral flow or in cases of subtotal occlusion), the myocardial metabolic demands, and the duration of coronary occlusion [5, 6]. The extent of myocardial damage following STEMI can be accurately assessed by cardiac magnetic resonance (CMR) utilizing late gadolinium enhancement (LGE) [7]. Nevertheless, LGE assessment in the acute phase post-AMI often overestimates the infarct size, whereas the exact assessment that is more precisely related to the amount of necrotic myocardium “or the final IS” needs to perform the LGE assessment several weeks after the index AMI [8] mitigating its advantage in guiding therapeutic strategies.

Myocardial strain is a quantitative index based on measuring myocardial deformation during a cardiac cycle [9]. One of the major tools for detecting changes in myocardial strain is speckle tracking echocardiography (STE) [10]. In the few past years, three-dimensional (3D) STE was introduced with a potential advantage to overcome the inherent shortcomings of two-dimensional (2D) STE. Therefore, it seemed quite intriguing to explore the performance of baseline strain parameters measured by 2D and 3D STE among other clinical parameters in predicting the final IS in a cohort of STEMI patients undergoing pPCI.

## Methods

### Study population

This single-center prospective observational study included 80 STEMI patients presenting to a tertiary center in Egypt through November 2018 to December 2019 for pPCI. Key inclusion criteria were patients’ age ≥ 18 years and first-time STEMI with symptoms onset less than 24 h. STEMI diagnosis was founded according to the 4th universal definition of MI [11]. The key exclusion criteria included prior STEMI (by history or subsequent imaging), receiving fibrinolytic therapy and referral for pharmaco-invasive or rescue PCI, presentation with cardiogenic shock, coronary anatomy not amenable for primary PCI and requiring urgent coronary artery bypass graft (CABG) surgery, angiographic evidence of unsuccessful reperfusion defined as thrombolysis in myocardial infarction (TIMI) grade < 2, and recognized contraindications to CMR. All patients signed an informed consent, while the study protocol and methodology were reviewed and approved by the institutional ethics committee.

### Baseline evaluation

All eligible STEMI patients underwent a focused review of demographic characteristics, risk profiles of coronary artery disease (CAD), and history of co-morbidities. Previous history of CAD was defined as reporting previous angina (or equivalent symptoms) prompting anti-ischemic medical therapy or elective coronary revascularization. Family history suggestive of premature CAD was defined as having ≥ 1 first degree (or ≥ 2 second degree) relative with established diagnosis of CAD or coronary revascularization under the age of 55 years or 65 years for males and females, respectively [12]. Clinical assessment included evaluation of vital signs, Killip class, precordial auscultation, and evaluation of signs of adequate peripheral perfusion. Weight and height were assessed, and body mass index (BMI) was calculated as (weight)/(height)^2^ in kilogram/meter^2^). Obesity was defined as a BMI ≥ 30. A 12-lead electrocardiogram (ECG) for establishing STEMI diagnosis and blood sampling for laboratory investigations were done upon admission. The maximum ST segment elevation (in millimeters) and the index lead showing the maximum elevation were recorded. Time delays until the pPCI were meticulously audited and tabulated. Total ischemic time represented the time from symptoms onset to restoration of IRA patency.

### Primary PCI

The procedure was performed according to the standard technique for coronary angiography and PCI. Performing thrombus aspiration and/or administering glycoproteins IIb/IIIa inhibitors (GPi) were selectively considered in cases with heavy thrombus burden or suboptimal final thrombolysis in myocardial infarction (TIMI) flow according to the operator’s discretion. The IRA (left anterior descending (LAD), right coronary artery (RCA), or left circumflex artery (LCx)) was identified and tabulated. Presence of severe lesions in other coronary vessels (defined as angiographically showing > 70% diameter stenosis) was also registered. Of note, the initial TIMI flow grade, the final TIMI flow grade, and the final TIMI myocardial blush grade (MBG) were registered according to the standardized definitions [13]. After pPCI completion by 30 min, another 12-lead ECG was performed, where the residual ST segment elevation in the index lead post-pPCI was registered. Also, second and third sets of cardiac troponins I (cTnI) were assessed after 8 and 16 h from the procedure, labeling the highest peri-procedural cTnI level (the highest value through the 3 sets).

### Echocardiographic examination

Patients underwent baseline transthoracic echocardiographic study within 48 h of admission using the xMATRIX X5-1 phased array sector probe (1–5 MHz) of the Philips EPIQ 7 machine (Philips Medical Systems, Andover, MA, USA). Digital 2D loops were acquired from basal, mid-, and apical parasternal short-axis views, and apical four-chamber, two-chamber, and long-axis views. The frame rate was adjusted at ≥ 50 frames per second during image acquisition.

Three-dimensional echocardiography images were acquired using the same probe and echocardiography machine. Images were acquired from the apical window, taking care to include the entire LV cavity within the pyramidal scan volume. The dataset was acquired over 4 consecutive cardiac cycles during a short breath-hold to minimize stitching artifacts.

For strain assessment, the digitally stored clips were analyzed offline using the TomTec software (TomTec-Arena, version 2.30, TomTec, Unterschleißheim, Germany]). For each of the three apical views, the operator manually identified three points: two on each side of the mitral valve and a third at the apex of the left ventricle. The software automatically detects the endocardium at end-systole and tracks myocardial motion during the entire cardiac cycle and then automatically calculates the end-systolic longitudinal and radial strain for each segment in a seventeen-segment LV model.

**Fig. 1:**
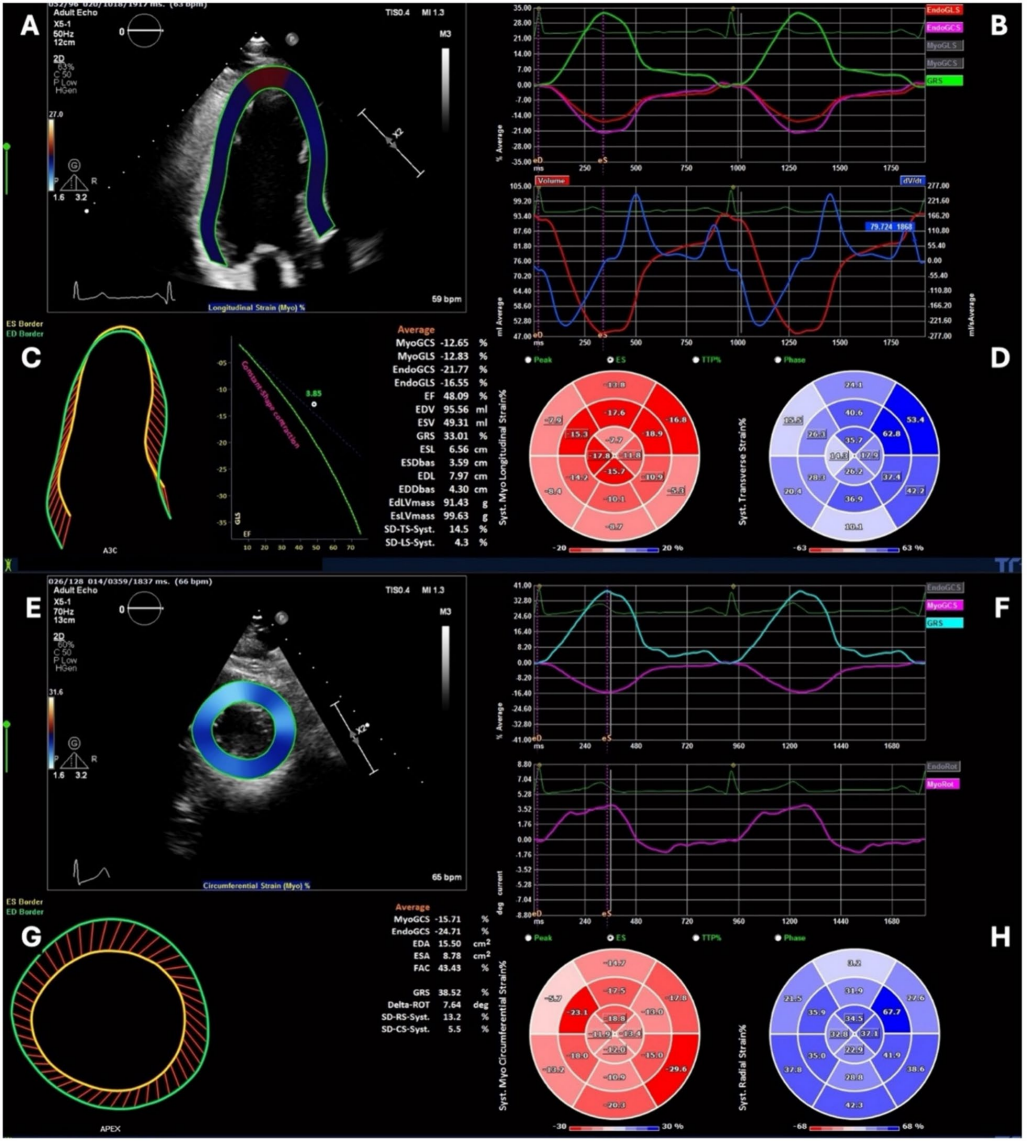
Two-dimensional strain acquisition and analysis. In panel **A**, the endocardial and epicardial contours are marked at the end systolic phase; then, the software automatically traces the defined contours throughout the cardiac cycle. Panel **B** shows the global strain curves. Panel **C**, displays the global LV values, and panel **D** shows the color-coded bull’s eye for both the longitudinal and the radial segmental strain values. In panel **E**, the endocardial and epicardial contours are marked at the end-systolic phase on each of the basal, mid-ventricular, and apical short-axis slices; then, the software automatically traces the identified contours throughout the cardiac cycle. Panel **F** shows the global strain curves. In panel **G**, it displays the global LV values and panel **H** shows the color-coded bull’s eye for the circumferential segmental strain values

**Fig. 2:**
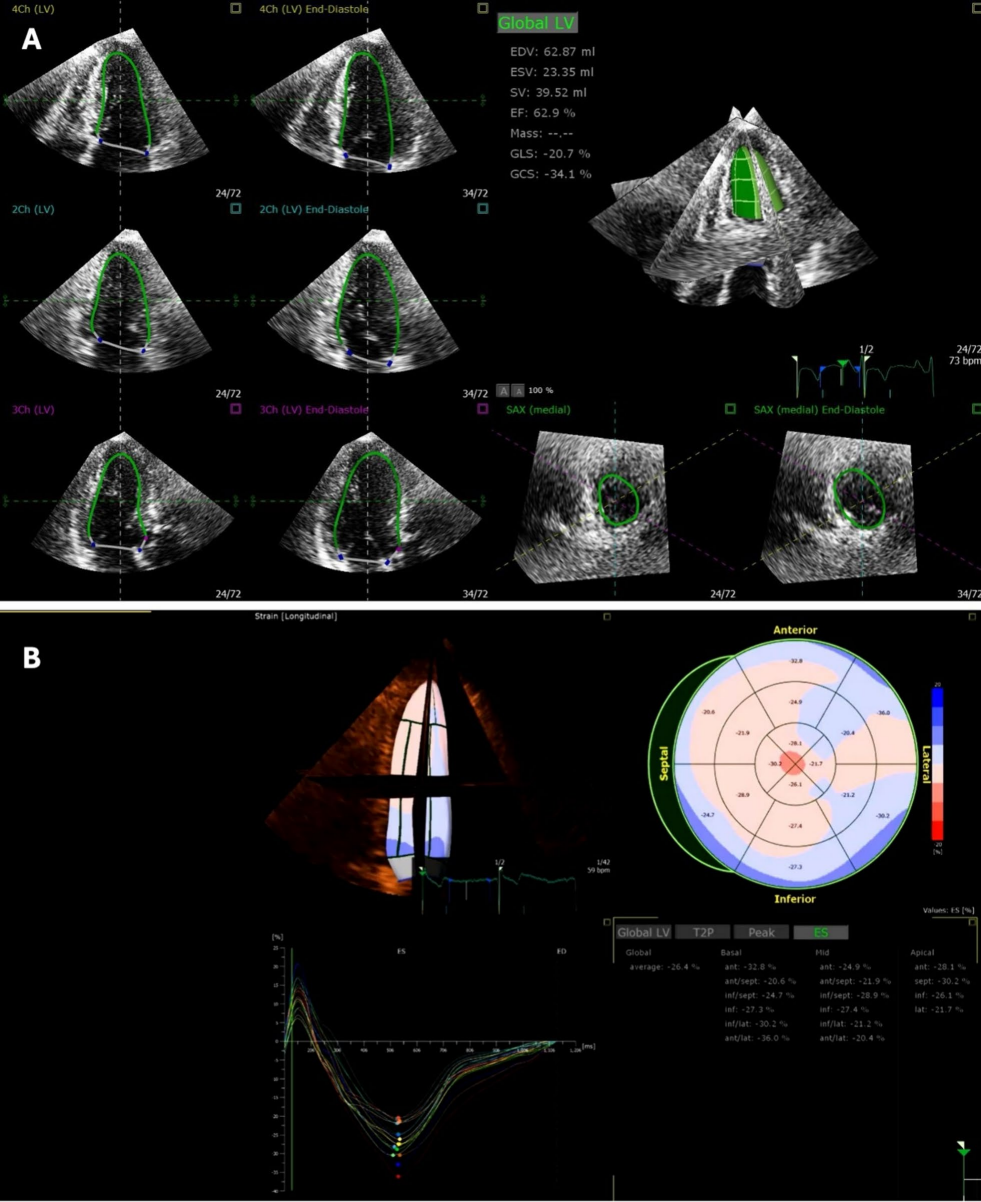
Three-dimensional strain acquisition and analysis. In Panel **A**, automatic tracking of the endocardial and epicardial contours in the end-systolic and end-diastolic 3D frames. Panel **B** shows the 3-dimensional echocardiography longitudinal strain results

After manual verification and approval of the tracing, the global longitudinal strain (GLS) and global radial strain (GRS) are automatically calculated by averaging the local strains along the entire LV. The software provides strain curves for the 16 myocardial segments (excluding the apical cap). Using a similar process for the short-axis views, the software generates end-systolic circumferential strain for each myocardial segment and hence the global circumferential strain (GCS). Because of the inherent significant inter- and intra-observer variability in the assessment of GCS by 2D, the investigators of this study planned to rely on 3D-derived GCS in the final analysis. End-systole was identified by the peak of the T wave while end-diastole coincided with the peak of the QRS complex. Figures 1 and 2 illustrate an example of the 2D and 3D strain analysis.

### Cardiac magnetic resonance

**Fig. 3:**
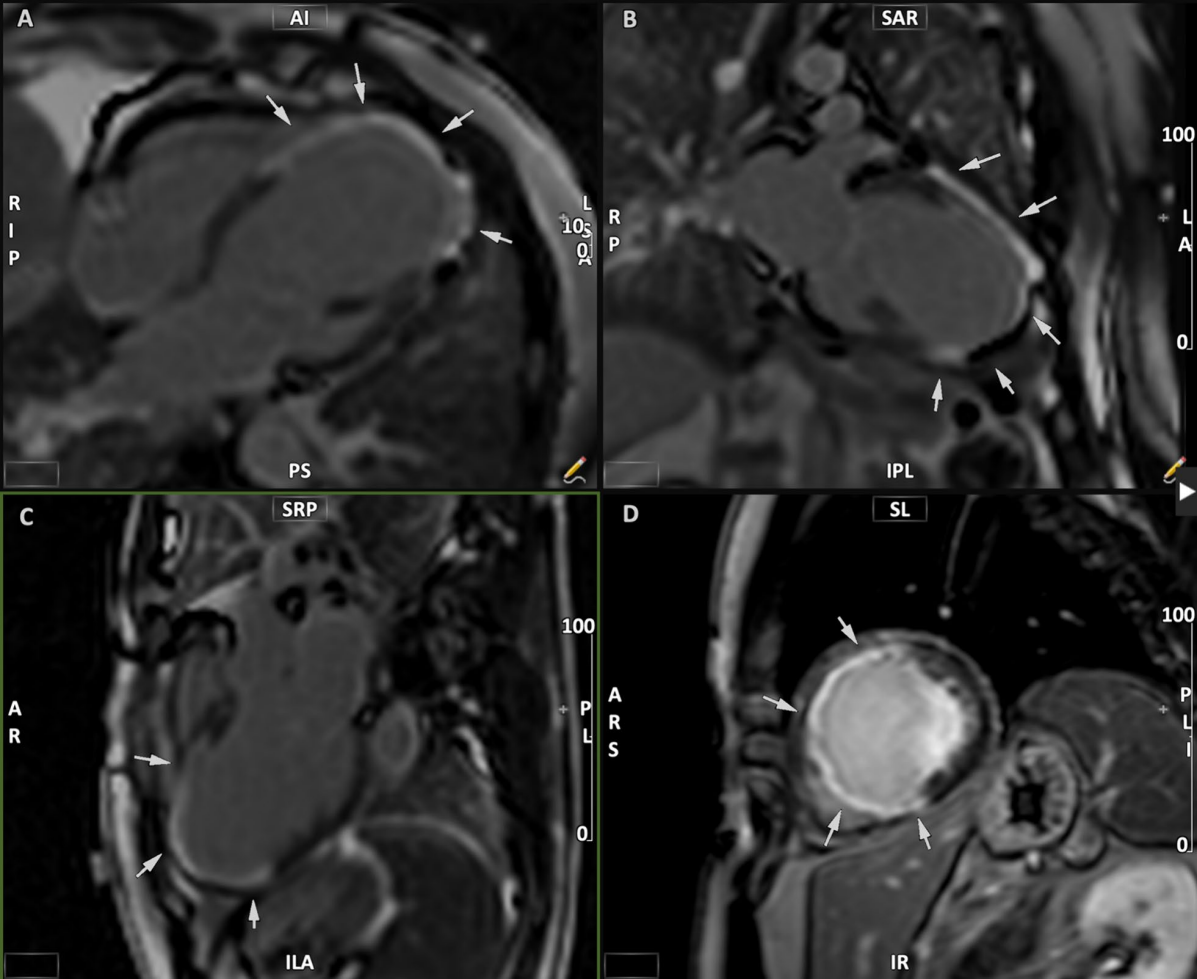
Evaluation of the final IS by LGE in CMR imaging. LGE evaluation at 3-month follow-up CMR to assess final IS. Panels **A**–**D** show 4-, 2-, 3-chamber and SAX views, respectively. White arrows point to the scarred myocardium in mid-to-apical anterior, mid-to-apical anteroseptal, and whole apical segments identified by delayed hyper-enhancement. *CMR* cardiac magnetic resonance, *IS* infarction size, *LGE* late gadolinium enhancement, *SAX* short axis

Each patient underwent CMR 3 months after the index pPCI. Studies were performed on a 1.5 Tesla CMR unit (AERA, Siemens Medical System, Erlangen, Germany) using ECG triggering and a cardiac-dedicated phase-array coil. For the assessment of LV volumes and function, steady-state free precession (SSFP) breath-held cine images were acquired in the following orientations: vertical long axis, horizontal long axis, and short axis. Standard parameters included repetition time/echo time of 3.6/1.8 ms; flip angle of 50°–70°; slice thickness of 6 mm; matrix of 160 × 256; field of view of 350–400 mm; and temporal resolution of 25 ms. The set of short-axis images was adjusted to encompass the entire LV, with a between-slices gap of 2 mm. For delayed enhancement imaging, a dose of 0.15 mL of gadolinium per kilogram of body weight was administered and images were acquired 10 min after contrast injection. The sequence used was a segmented inversion recovery gradient echo pulse sequence using the same image orientations as the cine images. Scan parameters were: TR/TE 4.01/1.25 ms, flip angle 15°, matrix 208 × 256, and voxel size 1.6 × 1.3 × 5 mm^3^. The T1 was adjusted to achieve optimal nulling of the myocardial signal. IS was determined using the full width at half maximum (FWHM) technique, which uses half the maximal signal within the scar as the threshold. In each slice, endocardial and epicardial contours were manually drawn and a region of interest was drawn around the infarct. All images were analyzed offline using the standard operational procedures in adherence to the published recommendations from the Society of Cardiovascular Magnetic Resonance [14]. Figure 3 demonstrates the assessment of the final IS by CMR LGE examination.

### Statistical analysis

All data were analyzed using the R statistical package version 4.2.0, with a two-tailed *P* value < 0.05 indicating statistical significance. Normally distributed numerical values were reported as mean ± standard deviation (SD), or median (25th- and 75th percentile). The assumption of normality was tested using histograms, quantile plots, and the Shapiro–Wilk test. Categorical variables were presented as counts and percentages. Univariable linear regression analysis was used to predict the final IS. Beta coefficients (representing the regression coefficients) and the 95% confidence intervals (CI) were reported for each variable, where positive and negative signs denote a direct or inverse relationship with the dependent variable (final IS), respectively. As we were particularly interested in exploring the prognostic value of 2D and 3D LVEF and GLS, we constructed an individual multivariable regression model for each of these parameters while adjusting for the significant clinical and angiographic predictors from the univariable analysis.

## Results

### Recruitment and pPCI

The study recruited 80 eligible STEMI patients, but at the time of CMR, 4 patients had uncontainable claustrophobia and were excluded, while 76 patients were included in the final analysis. The mean (SD) age was 54.1 ± 10.9 years, 84% were males, 25% were diabetics, 26% were hypertensives, 71% were current smokers, 82% had known history of dyslipidemia, 18% had family history of premature CAD, while 7% had history of previous CAD.

The mean time from symptoms onset to first medical contact was 170 min, while the mean total ischemic time was 310 min.

Anterior, inferior, and isolated lateral AMI occurred in 44 (58%), 31 (41%), and 1 (1%), respectively. Among the inferior MI, 14 (45%) had RV infarction and 4 (13%) had concomitant posterior wall infarction. The standard vascular access was radial artery (in 79% of cases), unless deemed unfeasible; then, femoral arterial access was utilized. Table 1 details the baseline, pPCI, and echocardiographic data of the whole study cohort.

**Table 1:** Baseline characteristics and pPCI data for the whole study cohort

Variable	Distribution
*Clinical parameters*	
Age (years)	54.1 ± 10.9
Male sex	64 (84%)
BMI (kg/m^2^)	27.9 ± 4.5
Obese	24 (32%)
Current smoker	54 (71%)
Hypertension	20 (26%)
Type II diabetes mellitus	19 (25%)
Dyslipidemia	60 (82%)
Known previous CAD	7 (9%)
Family history of premature CAD	14 (18%)
Previous ischemic stroke	3 (4%)
Admission heart rate (beat/minute)	87 (79, 98)
Admission systolic blood pressure	140 (130, 150)
ST elevation at baseline (millimeters)	3 (2, 5)
ST elevation post-PCI (millimeters)	1 (0, 2)
Pain to device (min)	310 (210, 432.5)
Total procedure time (min)	47.5 (30, 60)
Use of thrombectomy	34 (45%)
Use of glycoprotein IIb/IIIa inhibitors	44 (58%)
Contrast media volume	160 (130, 215)
Highest peri-procedural troponin	7.8 (2.9, 21.5)
*Angiographic parameters*	
Infarct related artery	
Left anterior descending	45 (60%)
Right coronary artery	26 (35%)
Left circumflex artery	5 (5%)
Baseline TIMI flow	
TIMI 0	56 (74%)
TIMI 1	9 (12%)
TIMI 2	11 (14%)
Final TIMI flow grade	
TIMI 2	6 (8%)
TIMI 3	70 (92%)
Final TIMI MBG	
MBG 0	6 (7%)
MBG 1	25 (33%)
MBG 2	27 (36%)
MBG 3	18 (24%)
Elective revascularization of other lesions after the index procedure	18 (24%)
*Baseline echocardiographic parameters*	
Wall motion score index	1.7 ± 0.3
Ejection fraction by 2D	45.0 ± 11.3
GLS 2D STE	− 8.8 (− 13.6, − 7.0)
GRS 2D STE	26.2 (19.8, 39.4)
Ejection fraction by 3D	45.7 ± 8.8
GLS 3D STE^†^	− 13.3 (− 18.7, − 10.0)
GCS 3D STE^†^	− 20.8 (− 23.5, − 16.0)
GRS 3D STE	29.3 (22.7, 35.9)

Data represented as mean ± standard deviation, median (25th, 75th percentiles), or frequency (%) as appropriate

*2D* two-dimensional, *3D* three-dimensional, *BMI* body mass index, *CAD* coronary artery disease, *GCS* global radial strain, *GLS* global longitudinal strain, *GRS* global radial strain, *MBG* myocardial blush grade, *pPCI* primary percutaneous coronary intervention, *STE* speckle tracking echocardiography, *TIMI* thrombolysis in myocardial infarction

^†^Retaining the numerical negativity, i.e., a GLS of − 18 is smaller numerically than − 10 but represents a better strain function

### Predictors of final infarct size

**Fig. 4:**
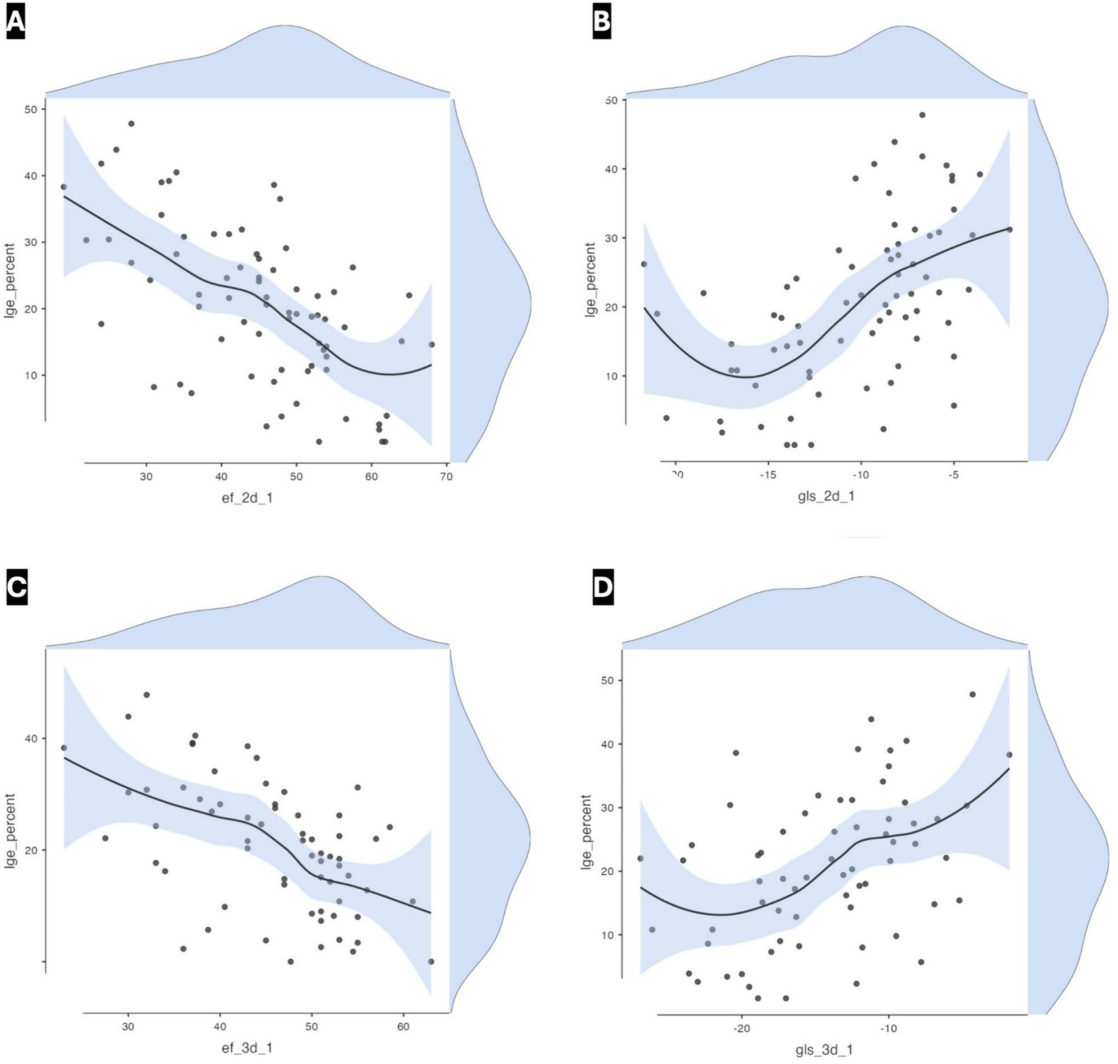
Regression lines with scatterplots for 2D- and 3D echocardiographic parameters in predicting final IS by LGE. Scatterplots for 2D-EF, 2D-GLS, 3D-EF, and 3D-GLS in panels **A**–**D**, respectively, demonstrating the regression lines and with shadowing of the reading densities for LGE on the Y axis (to the right) and of the predictor variable on the X axis (above) for each corresponding variable. *2D* two-dimensional, *3D* three-dimensional, *EF* ejection fraction, *GLS* global longitudinal strain, *IS* infarct size, *LGE* late gadolinium enhancement

Among the baseline clinical and angiographic characteristics, predictors of the final IS in the univariate regression analysis were BMI, hypertension, both the baseline and the post-procedural (residual) ST elevation, anterior and inferior locations of MI, presence of multi-vessel CAD, administration of GPi, use of thrombectomy, baseline TIMI flow grade, and final myocardial blush grade, while among the 2D- and 3D echocardiographic and STE parameters, EF, GLS, and GRS by both modalities, 3D-STE-derived GCS, and the 2D-derived wall motion score index (WMSI) were all significant predictors for the final IS. Univariate analysis data are detailed in Tables 2 and 3 and Fig. 4.

**Table 2:** Univariate regression analysis to assess the predictive ability of baseline characteristics for final infarct size

Variables	Beta coef	95% CI	*P* value
BMI (kg/m^2^)	− 0.71	− 1.3, − 0.13	0.017
Hypertension	− 8.0	− 14, − 2.1	0.008
Admission heart rate	0.21	0.04, 0.38	0.019
Admission systolic blood pressure	− 0.1	− 0.2, 0.01	0.068
Pain to device time	0.01	0.00, 0.01	0.21
Baseline ST segment elevation	1.7	0.83, 2.6	< 0.001
Post-pPCI ST segment elevation	3.7	1.7, 5.7	< 0.001
Anterior myocardial infarction	9.0	3.9, 14	< 0.001
Inferior myocardial infarction	− 8.7	− 14, − 3.5	0.001
Multi-vessel CAD	5.0	2.1, 7.9	0.001
Highest peri-procedural troponin	0.53	0.14, 0.93	0.008
Use of glycoprotein IIb/IIIa inhibitors	8.4	3.3, 14	0.002
Use of thrombectomy	6.8	1.6, 12	0.011
Baseline TIMI flow grade	− 4.4	− 6.9, − 1.9	< 0.001
Final TIMI flow grade	− 7.7	− 17, 1.8	0.11
Final TIMI MBG	− 4.5	− 7.3, − 1.6	0.002

*BMI* body mass index, *CAD* coronary artery disease, *CI* confidence interval, *coef* coefficient, *MBG* myocardial blush grade, *pPCI* primary percutaneous coronary intervention, *TIMI* thrombolysis in myocardial infarction grade

**Table 3:** Univariate regression analysis to assess the predictive ability of baseline 2D and 3D echocardiographic and strain parameters for final infarct size

Variables	Beta coef	95% CI	*P* value
2D-EF (%)	− 0.62	− 0.82, − 0.43	< 0.001
2D-WMSI	24	17, 30	< 0.001
2D-GLS^†^ (%)	1.4	0.85, 1.9	< 0.001
2D-GRS (%)	− 0.25	− 0.40, − 0.09	0.002
3D-EF (%)	− 0.73	− 1.0, − 0.44	< 0.001
3D-GLS^†^ (%)	0.92	0.46, 1.4	< 0.001
3D-GCS^†^ (%)	1.0	0.55, 1.5	< 0.001
3D-GRS (%)	− 0.73	− 1.0, − 0.44	< 0.001

*2D* two-dimensional, *3D* three-dimensional, *CI* confidence interval, *coef* coefficient, *EF* ejection fraction, *GCS* global circumferential strain, *GLS* global longitudinal strain, *GRS* global radial strain, *WMSI* wall motion score index

^†^Retaining the numerical negativity, i.e., a GLS of − 18 is smaller numerically than − 10 but represents a better strain function

### Multivariate regression analysis to predict IS

Four multivariate regression analysis models adjusting for the admission heart rate, post-pPCI ST segment elevation, the highest peri-procedural cTnI value, and inferior STEMI (vs. anterior) were designed for each of the 2D- and 3D LVEF and GLS, respectively. The residual ST-elevation was consistently an independent predictor for the final IS, while the highest periprocedural troponin proved an independent predictor in 3 out of the 4 models. In the corresponding models, 2D LVEF, 3D LVED, and the 2D GLS proved to be significant independent predictors for the final IS, while the 3D GLS fell short of significance with a *P* value of 0.052. The multivariate regression models are demonstrated in Table 4.

**Table 4:** Multivariate regression analysis to identify independent predictors for final infarct size

Predictor	Beta coef	95% CI	*P* value
*(a) Model incorporating the baseline 2D-LVEF*			
OA heart rate	0.04	− 0.11, 0.19	0.58
Post-pPCI ST elevation	2.86	0.86, 4.87	0.006
Highest peri-procedural troponin	0.41	0.10, 0.72	0.009
Inferior STEMI	− 0.17	− 7.13, 3.74	0.54
2D-LVEF	− 0.54	− 0.76, − 0.33	< 0.001
*(b) Model incorporating the baseline 2D-GLS*			
OA heart rate	0.1	− 0.06, 0.25	0.23
Post-pPCI ST elevation	2.88	0.6, 5.2	0.014
Highest peri-procedural troponin	0.37	0.02, 0.71	0.037
Inferior STEMI	− 1.54	− 8.31, 5.23	0.65
2D-GLS	0.99	0.3, 1.7	0.006
*(c) Model incorporating the baseline 3D-EF*			
OA heart rate	0.03	− 0.16, 0.21	0.78
Post-pPCI ST elevation	3.9	1.61, 6.19	0.001
Highest peri-procedural troponin	0.37	0.05, 0.69	0.026
Inferior STEMI	− 1.28	− 7.04, 4.49	0.66
3D-LVEF	− 0.58	− 0.87, − 0.29	< 0.001
*(d) Model incorporating the baseline 2D-GLS*			
OA heart rate	0.07	− 0.13, 0.27	0.49
Post-pPCI ST elevation	3.81	1.28, 6.35	0.004
Highest peri-procedural troponin	0.33	− 0.03, 0.69	0.068
Inferior STEMI	− 0.29	− 6.87, 6.3	0.93
3D-GLS	0.54	0, 1.1	0.052

*2D* two-dimensional, *3D* three-dimensional, *coef* coefficient, *CI* confidence interval, *EF* ejection fraction, *GLS* global longitudinal strain, *OA* on-admission, *pPCI* primary percutaneous coronary intervention, *STEMI* ST segment elevation myocardial infarction

## Discussion

In this study, 80 eligible primary PCI patients had baseline 2D and 3D standard echocardiography and STE; then, after 3 months from the index STEMI, they underwent CMR with LGE for accurate evaluation of the final IS. Among the clinical characteristics, systemic arterial hypertension, BMI, inferior STEMI, the initial TIMI flow grade, and the final MBG were negative predictors, while the admission heart rate, the baseline ST segment elevation, the post-pPCI ST segment elevation, the highest peri-procedural cTn, anterior STEMI, use of thrombectomy, use of GPi, and multi-vessel CAD were positive predictors for the final IS. Evaluating the predictive performance of 2D- and 3D baseline echocardiographic and STE assessments demonstrated that LVEF and GLS were significant predictors for the final IS in univariate regression analysis. In the multivariate regression analysis, the post-pPCI ST segment elevation, the highest peri-procedural cTnI level, the baseline EF (both 2D and 3D), and the baseline 2D-GLS were independent predictors for the final IS.

Acute STEMI remains an overwhelming cardiac emergency with serious sequelae on survival, morbidity, and quality of life [15]. An even larger aftermath occurs in developing countries (like in the Middle East region) where a substantial proportion of STEMIs affects young patients, hence impacting the manpower and the national productivity [12]. Primary PCI represents the ultimate management of acute STEMI by timely restoring patency of the IRA in order to minimize myocardial injury, preserve LV function, and reduce morbidity and mortality [15]. Nevertheless, occasionally the myocardial salvaging is suboptimal despite an angiographically successful pPCI, either because of prolonged ischemic time, reperfusion injury, microvascular obstruction, or a combination of several contributors [16, 17].

While reducing mortality as a hard endpoint represents the most meaningful outcome to evaluate the reperfusion efficacy, the low event rate in contemporary practice necessitates large sample sizes and extended years of follow-up to appreciate the impact of any investigational advent [15, 18]. Thereby, parameters with proven correlation to mortality that are discernable within a convenient time frame represent a vital requirement [15, 19]. Among several parameters, CMR-derived final IS showed excellent correlation to mortality and hence became an approved endpoint for post-AMI prognostication [20]. It was shown that the final IS quantified by CMR-LGE was a stronger predictor for all-cause mortality compared to LVEF and/or LV volumes in post-AMI patients [21].

However, early CMR assessment shortly after successful pPCI often overestimates the myocardial injury, and several weeks are required for the complete resolution of the ischemic and reperfusion myocardial injury/edema to allow for precise quantification of IS [22]. Thus, identifying early predictors for the final IS can be of extreme value allowing to tailor therapeutic strategies in light of the individualized patients’ risk. Hence, this study opted for seeking the early clinical and echocardiographic predictors for final IS in a cohort of acute STEMI patients who underwent successful pPCI.

Our results demonstrated that higher admission heart rate was a significant positive predictor, while hypertension was a significant negative predictor for final IS. Other studies recruiting STEMI patients who underwent successful pPCI had shown that higher admission heart rate was associated with a reduction in salvaged myocardium and an increase in final IS [23, 24]. Similar findings were consolidated from animal models evaluating experimental coronary occlusion, where higher heart rates were correlated with more extensive myocardial damage, plausibly because it increases the myocardial oxygen demands [25]. Upon presentation with AMI, it had been shown that a higher heart rate and a lower systolic blood pressure (reflecting the cardio-circulatory stress and hemodynamic reserve, respectively), were associated with subsequent heart failure and death [26]. Furthermore, the quotient of heart rate/systolic blood pressure comprising the simple and conventional shock index (SI) demonstrated excellent correlation with short- and mid-term outcomes after STEMI in another study [26].

BMI in our results was a negative predictor for final infarct size (the more the BMI, the less the IS). In another study that had recruited 225 STEMI patients treated with pPCI, they found that BMI ≥ 28 kg/m^2^ was independently associated with a lower risk of microvascular obstruction and a smaller AAR and subsequently a smaller final IS [27]. Although obesity is associated with a higher risk of CAD, the inverse relation between BMI and final IS may denote an obesity paradox similar to that observed in heart failure patients [28].

Anterior infarction was a predictor for a larger final infarct size in contrast with inferior infarction which predicted smaller infarctions. Anterior STEMIs caused larger myocardial scars compared to inferior ones in another study that recruited 136 consecutive STEMI patients [29]. Arguably, the LAD has the largest territory of the LV myocardial mass with minimal overlap with other coronary distributions, while the inherent overlap between the RCA and LCx branching may help to mitigate the resultant myocardial damage if one of them got acutely occluded.

In our study, higher magnitudes of ST elevation (represented in mm), both in the baseline (pre-procedure) and the post-pPCI (in the ECG after 30 min from procedure completion), were significant predictors for larger final IS. Contrary to the dogmatic belief that > 50% normalization of the ST elevation is a marker for successful reperfusion, there is growing evidence that the absolute residual ST elevation after pPCI is an independent predictor for clinical outcomes, including survival and reinfarction at 30 days and 1-year [30]. In another study recruiting anterior STEMI patients, the investigators found that post-pPCI ST segment elevation less than 3.25 mm had 70% sensitivity and 82% specificity (AUC = 0.86) for predicting CMR infarct size less than 16% at 1 year [31]. In another study, the absolute residual ST segment elevation (in mm) in the index lead (with the highest baseline elevation) had better performance in predicting cardiac function recovery and final IS by CMR, compared to either percentage of resolution in the index lead, or percentage of resolution of the sum of all affected leads [32]. The utility of other ECG parameters to predict the efficiency of myocardial reperfusion after pPCI (which would rather be associated with the reduction in final IS) had been sought in different studies. These include QT dispersion, heart rate variability, and signal-averaged ECG; however, consistent data about a solid predictive performance are lacking [33–35].

We have recognized that the highest cTnI value within the first 24 h peri-procedure was an independent predictor for the final IS by CMR at 3 months. The association between the final IS and single-point sampling of cTnI was also investigated in another study, where the investigators measured cTnI 24 and 48 h after admission then CMR at 5 days and 4 months post-pPCI. There was a robust correlation between the extent of LGE by CMR and cTnI sampled at 24 h (*r* = 0.66 (5 days) and *r* = 0.63 (4 months)) and 48 h (*r* = 0.67 (5 days) and *r* = 0.65 (4 months)). In the multiple regression analysis, cTnI proved to be an independent predictor for the final IS even with adjustment for the early infarct size (LGE by CMR on the 5th day) [36].

We noticed that the use of thrombectomy and administration of GPi were significant predictors for larger infarct size. Notably, this does not mean that the use of thrombectomy or GPi led to an increase in the final IS, simply because they were selectively used upon operator discretion and hence can be considered as a surrogate for their indication which was large thrombus burden or no-reflow complication. On the contrary, studies randomizing all STEMI patients with large thrombus burden to either aspiration thrombectomy plus PCI or PCI only showed that thrombus aspiration resulted in significantly less microvascular obstruction (MVO) and smaller MI size by CMR at 6 months [37]. Therefore, it is generally believed that when appropriately indicated, thrombus aspiration in selected patients is potentially beneficial to lessen microvascular obstruction and improve myocardial salvage, yet with the potential risk of increased cerebrovascular strokes [38].

Our results demonstrated that baseline TIMI flow and final MBG were significant predictors for the final IS. Clearly, if baseline TIMI flow is > 0, the maintained antegrade flow to the jeopardized myocardium would mitigate the extent of both the ischemic and the subsequent reperfusion injuries. Although difficult to ascertain if it was partial spontaneous recanalization or was subtotal occlusion from the outset, it had been demonstrated in several studies that STEMI patients presenting with baseline TIMI flow ≥ 1 often encounter smaller final IS [22, 39]. Interestingly, these studies similar to our results found that the final TIMI flow was not significantly correlated with the final IS, which could be reasoned because the final TIMI flow was grade III in the majority of pPCI patients.

Contrarily, we have found that the final TIMI MBG has proved to be a significant prognosticator after successful restoration of the IRA patency. Similarly, in a study that enrolled 924 STEMI patients, they found that the “enzymatic infarct size” was larger in patients with final MBG 0 or 1 compared to those with MBG 2 and 3 (1437 ± 2388 vs. 809 ± 1672, *P* = 0.001) [40]. The authors of that study strongly recommended that the angiographic definition of successful reperfusion should strictly entail a TIMI 3 flow coupled with MBG 2 or 3 [40]. In another study that enrolled 1213 patients with STEMI and TIMI grade-3 flow after pPCI, MBG ≤ 2 was associated with poorer myocardial salvage, larger infarct, and higher 5-year mortality than observed in patients with MBG = 3 [19].

In the present study, the LVEF, GLS, and GRS (both by 2D and 3D) as well as the GCS (by 3D) proved to be significant predictors for the final IS in the univariate regression analysis.

Among the baseline characteristics, the post-PCI ST elevation and the highest peri-procedural cTnI were the independent predictors, while in the corresponding models for echocardiographic parameters, the baseline 2D-LVEF, 3D-LVEF, and 2D-GLS (but not the 3D-GLS) proved to be significant independent predictors for the final IS.

Although the evidence supports a superior impact of final IS (quantified by CMR-LGE) over the EF regarding mortality in STEMI patients, a recent literature review that included 27 studies suggested that EF still holds a very robust correlation with post-STEMI prognosis and mortality and thus can serve as a useful surrogate in future STEMI trials of therapies intended to improve ventricular function and mortality [41]. This underscores the importance of accurate assessment of EF in STEMI patients using multimodalities, especially 3D techniques to ensure meticulous evaluation and minimization of geometric assumptions.

It had been established that assessment of LV function after STEMI is often challenged by the compensatory hyperkinesis in the noninfarct territory and by the geometric assumptions inherent in the traditional 2D imaging, particularly with the disordered contour of the LV following MI [42]. Such limitations sparked the introduction of the wall-motion score index and 3D LVEF assessments [42, 43]. Currently, 3D imaging is bridging the gap of the fallacious geometric assumptions; however, the imaging quality of the present technologies is still in its dawn. The suboptimal spatial resolution in 3D imaging frequently leads to faulty inclusion of the LV trabeculae within the myocardial tracing, hence underestimating LV volumes [44]. Moreover, temporal irregularities (for example provoked by bundle branch block) continue to cause fallacious assessments, especially in post-AMI patients, especially if have uncontrolled pulse rates, thus resulting in a higher likelihood of stitching artifacts.

In the present study, we found that baseline EF assessed by 2D and 3D echocardiography predicts final IS at 3 months assessed by CMR-LGE, both in univariate and multivariate regression analysis.

In concerns to the baseline STE, in univariate analysis, our results demonstrated that baseline global strain values were significant predictors for the final IS; however, only 2D- and not 3D-derived GLS preserved statistical significance after adjustment for clinical and ECG parameters in the multivariate regression analysis models.

In a recent CMR study that recruited 100 patients after their first anterior STEMI successfully treated with pPCI, the investigators evaluated the baseline 2D-GLS within 48h of admission and then subsequently evaluated the final IS 3 months later using LGE-CMR. A large infarct was dichotomously defined as a scar in ≥ 20% of the LV myocardium. Baseline GLS showed excellent correlation with the final IS (*r* = − 0.840, *P* < 0.001), while ROC curve analysis showed a cut-off point of GLS (− 13%) identifying a large final IS with a sensitivity and specificity of 66.7% and 88.4%, respectively (AUC = 0.85) [45]. In another study that assessed the role of 3D-STE in detecting microvascular obstruction and myocardial impairment after the first STEMI in 100 subjects, the investigators demonstrated that all the 3D strain parameters in the infarcted segments were significantly impaired compared to the non-infarcted segments. Moreover, the segmental area strain and radial strain were significantly associated with the diagnosis of transmural LGE (*P* < 0.01) [46].

There has been a recent controversy in the literature on the utility of the 3D-STE derived global strain values and if there was an advantage over the 2D-STE assessment. At least for the present times, the temporal and spatial resolutions are considerably lower in 3D than in 2D STE [47]. In several reports including a large systematic review that had included 11 studies and a total of 765 patients, the 2D-GLS was better correlated with the final IS (evaluated by CMR) compared to the 3D-GLS [48]. At least for the current technologies, it seems that 3D-STE is not advantageous over 2D-STE, where each modality has its limitations [47].

The fast pace in technical advancement in 3DE and 3D-STE is very promising to see soon the needed promotions for the suboptimal temporal and spatial resolution and to have standardization of 3D STE assessment across different vendors, where the present heterogeneity is another root for disagreements [49].

To summarize,

In this study, all strain variables measured by the two modalities (2D and 3D echocardiography) effectively predicted final IS in the follow-up CMR imaging. Acknowledging the strong association of the final IS with subsequent LV functional recovery and overall patients’ survival, seeking clinical, biomarkers, ECG, and imaging predictors for larger final IS can serve as a valuable tool for stratification to identify high-risk subgroups. Future work to test the application of aggressive disease-modifying strategies to improve the natural history of patients identified to be at higher risk is warranted.

### Limitations

The study was conducted in a single tertiary referral center, which may limit the generalizability of the results. Patients with uncontainable claustrophobia were unable to undergo the CMR examination and thus were excluded from the analysis of final IS and all subsequent correlates. Assessment of GCS by 2DE was found to be very challenging to acquire with adequate accuracy in a substantial proportion of patients due to sub-optimal echocardiographic window; thus, the investigators resorted to relying on the 3D assessment of this strain index.

## Conclusions

Final IS assessed by LGE-CMR at 3 months post-STEMI is significantly dependent on admission heart rate, STEMI location, baseline and post-PCI residual ST-elevation, highest peri-procedural cTnI values, as well as LVEF and all global strain indices assessed by both 2DE and 3DE. After adjustment for potential confounders in multivariate regression analysis, post-PCI residual ST-elevation, highest periprocedural cTnI, 2D-LVEF, 3D-LVEF, and 2D-GLS were independent predictors for final IS. These can serve as vital tools for a timely stratification of patients’ risk to guide the intensification of therapeutic strategies.

## Data Availability

These can be made available upon reasonable request from the corresponding author.

## Abbreviations

2D: 2-Dimensional
3D: 3-Dimensional
ACS: Acute coronary syndrome
AHA: American Heart Association
AHF: Acute heart failure
AMI: Acute myocardial infarction
ASCAD: Atherosclerotic coronary artery disease
ASE: American Society of Echocardiography
AT: Aspiration thrombectomy
BMI: Body mass index
BSA: Body surface area
CABG: Coronary artery bypass graft
CAD: Coronary artery disease
CCU: Coronary care unit
CI: Confidence interval
CIN: Contrast-induced nephropathy
CK-MB: Creatine kinase myocardial band
CMR: Cardiac magnetic resonance
CS: Circumferential strain
CTnI: Cardiac troponin I
CV: Cardiovascular
DAPT: Dual antiplatelet therapy
DES: Drug-eluting stent
DM: Diabetes mellitus
EACVI: European Association of Cardiovascular Imaging
ECG: Electrocardiogram
EDV: End-diastolic volume
EF: Ejection fraction
eGFR: Estimated glomerular filtration rate
ESV: End-systolic volume
GCS: Global circumferential strain
GDMT: Guideline-directed medical treatment
GLS: Global longitudinal strain
GRS: Global radial strain
HBA1c: Glycated hemoglobin
HF: Heart failure
HFrEF: Heart failure with reduced ejection fraction
HR: Heart rate
IRA: Infarct-related artery
LAD: Left anterior descending
LBBB: Left bundle branch block
LCX: Left circumflex
LDL-C: Low-density lipoprotein cholesterol
LGE: Late gadolinium enhancement
LV: Left ventricle
LVEDV: Left ventricular end-diastolic volume
LVEF: Left ventricular ejection fraction
LVESV: Left ventricular end-systolic volume
LVR: Left ventricular remodeling
LVR: Left ventricular remodeling
MACE: Major adverse cardiovascular events
MI: Myocardial infarction
MRA: Mineralocorticoid receptor antagonist
MRI: Magnetic resonance imaging
MVO: Microvascular obstruction
NF-KB: Nuclear factor-KB
NPs: Natriuretic peptides
NSTEMI: Non-ST elevation myocardial infarction
NT-pro-BNP: N terminal pro brain natriuretic peptide
NYHA: New York Heart Association
OAC: Oral anticoagulant
PCI: Percutaneous coronary intervention
pPCI: Primary percutaneous coronary intervention
PTCA: Percutaneous transluminal coronary angioplasty
RCA: Right coronary artery
RCTs: Randomized controlled studies
RS: Radial strain
RV: Right ventricle
RVFAC: Right ventricular fractional area change
STE: Speckle tracking echocardiography
STEMI: ST elevation myocardial infarction
STR: ST segment resolution
TAPSE: Tricuspid annular plane systolic excursion
TDI: Tissue Doppler imaging
TIMI: Thrombolysis in myocardial infarction
TLR: Target lesion revascularization
TTE: Trans-thoracic echocardiography
